# Childhood Cancer and the Family: A Pilot Proposal for Comprehensive Intervention at the Time of Diagnosis

**DOI:** 10.3390/healthcare10091790

**Published:** 2022-09-16

**Authors:** Marta Mira-Aladrén, Javier Martín-Peña, Gemma Sevillano Cintora, Antonio Celma Juste, Marta Gil-Lacruz

**Affiliations:** 1 Faculty of Social Sciences and Labor, University of Zaragoza, 50009 Zaragoza, Spain; 2 Association of Parents of Oncological Children of Aragon (ASPANOA), 50009 Zaragoza, Spain; 3 Faculty of Health Sciences, University of Zaragoza, 50009 Zaragoza, Spain

**Keywords:** childhood cancer, diagnosis, family impact, crisis intervention, social work

## Abstract

Childhood cancer has a great impact on children and their environment. To minimize this, countries such as Canada and the USA have protocols in the field of social work, although these are scarce in Europe and especially in Spain. This paper aims to develop a pilot protocol in Aragon (Spain) for the practice of onco-pediatric social work in one of the hardest moments: the diagnosis. For its elaboration, a previous study was carried out in three phases, which provided data on the disease and its impact on the family and children and a methodological basis for the intervention from social work, all considering the participation of the agents involved as a fundamental element. Variables have been identified that influence the impact on the family support network and its quality of life at the time of diagnosis of childhood cancer. In addition, different indicators have been explored, based on the reality of these families. Finally, a pilot proposal for a comprehensive family intervention protocol in the diagnosis of childhood cancer has been elaborated. This work is intended to be a guide for intervention and delimitation of quality standards to be considered when dealing with the diagnosis of childhood cancer.

## 1. Introduction

Cancer diagnosis has a great impact on the social imaginary because it is related to death and suffering, aggravated in the case of childhood cancer. This disease, considered serious in the health field, has the peculiarity of affecting a vulnerable and developing population. Family is a primary agent of health socialization [1,2,3], so it is essential to address the impact of childhood cancer on patients and their families, as it affects their future development.

It is pointed out how “the child constructs the meaning of their illness, understands what happens to them, through what parents think, feel, show... and how they act in the face of this illness” [1]. This meaning can affect adherence to treatment, the development of post-traumatic stress symptoms—present in 23% of cases [1]—or the acceptance of the after-effects. It is therefore advisable to analyze the reality of this disease and its repercussions, considering psychological and socio-economic and family aspects, in children and their families.

This approach can be provided by social work, which can contribute interesting results for the professionals involved in dealing with childhood cancer and its effects on patients and their families [2,4]. In this sense, within USA and Canada, through the Association of Pediatric Oncology Social Workers (APOSW), important efforts are underway at international level in the creation of protocols and standards of this type [4,5,6]. Despite the progress they represent, some studies on these standards have detected deficits in their implementation, such as the lack of staff, the difficulty of access to mental health, the lack of standardized and universal diagnostic and assessment tools, or the need for support for professionals, patients and families, especially in situations that arise from COVID-19 [5,6].

However, at the European level, and specifically in Spain, there is a scarcity of studies from social work, which constitutes an opportunity for research. The studies analyzed suggest that issues such as family reorganization, socio-economic support, the child’s environment, psychoeducation, counselling and information directly influence the resilience of those affected [5,7,8]. These studies, together with the results obtained in the literature review, favor the creation of categories of analysis in studies such as this one. Some of these categories are empowerment [7,8], perception of support/loneliness in the support network [7,8], psychological needs [5,8], or variables that influence the process [9,10].

Some of these issues are part of the functions of the onco-pediatric social worker [5,6,11], allowing a greater understanding of the situation, promoting comprehensive and person-centered care as an opportunity for empowerment. In addition, care must be comprehensive and interdisciplinary, addressing the needs that may arise in patients and their environment from all areas affected, all with the aim of improving the well-being of the person and their environment and minimizing the bio-psychological and socio-economic consequences of the disease [12].

In view of the role and functions of the onco-pediatric social worker and the limitations detected in research in this field [5,6], it seems necessary to seek criteria of quality and care applicable to social intervention at the time of diagnosis of childhood cancer. To this end, our general objective is to elaborate a pilot intervention protocol for the comprehensive care of families of children with cancer at the time of diagnosis in Aragon (Spain). Three specific objectives are pursued: (1) to explore the situation of those affected by childhood cancer; (2) to describe how children, their families and their close environment cope with the disease; and (3) to study possible indicators to generate interventions to be developed.

## 2. Materials and Methods

Our study, prepared together with the Association of Parents of Oncological Children of Aragon (ASPANOA), was carried out with a qualitative and participatory methodology that took a broad look at the reality of people affected by childhood cancer and their environment, through three phases: (1) analysis of secondary sources, (2) collection of qualitative data through interviews, and (3) implementation of a participatory process for the design of the intervention protocol, according to the results of the previous phases.

Phase 1, focused on objectives 1 and 2, consisted of a literature review using search engines and databases (e.g., Scopus, Web of Science), specialized publications of the National Cancer Institute (INC), the International Agency for Research on Cancer (IARC) and the World Health Organization (WHO). The keywords used individually and in combination were: “childhood cancer”, “onco-pediatric”, “diagnosis”, “family impact”, “crisis intervention”, “social work”, and “bad news”, in Spanish and English. A complementary search of existing resources and guides was conducted, mainly in Spain and the USA, using the following online resources: Spanish Federation of Parents of Children with Cancer [13] and its associations, the Health Departments of the Spanish Autonomous Communities and the US Department of Health. Finally; we used 40 publications in Spanish and 18 in English, from 2005 to 2020.

These results allowed us to find out that the area in which most research on onco-pediatric social work is carried out is North America, especially linked to the APOSW protocols and focused on the wellbeing of the family and the child as well as the professionals who care for them. They also served as a basis for generating the categories of analysis used in phase 2. Therefore, it can be stated that the literature review was selected as the methodological procedure because of the importance of having empirical information contrasted by health authorities and other sources of information.

For phase 2, the qualitative interview technique was chosen in order to delve deeper into the reality of those affected, and to involve different actors. Its objectives were:

1. To describe indicators based on the reality of onco-pediatric patients’ families.
2. To find out the impact of the diagnosis of the disease on the family’s social support network and their quality of life.
3. To infer possible indicators for analyzing interventions developed to date.

The interviews were conducted with a convenience sample selected according to the objectives and characteristics of the study. Five people were chosen according to a criterion of significance, considering the resources available. Finally, four interviews were conducted, by prior agreement, with professionals and affected families. Informant 1 could not be interviewed due to health problems.

In response to the interest in giving a voice to the families affected by the disease, empowering them and gathering their vision for a better intervention, we collaborated with ASPANOA, as a liaison with the families and professionals. They were also involved in the interview design, prior to its application, in order to minimize the harmful effects that this could have on the relatives interviewed. The ethical issue was prioritized over that of possible bias. Finally, the modifications made by the organization were minimal and mainly of a technical nature, so this decision did not affect the substance of the research.

The interviews were designed by two psychologists and a social worker. These tools were applied by the main researcher, a social worker, after supervision and advice from ASPANOA professionals, as mentioned above. In order to encourage the openness of the interviewees, the interviews were conducted in a comfortable space for the interviewees, the ASPANOA headquarters and the hospital where they work or volunteer. For the same purpose, the interviews lasted no longer than one hour. Finally, they were transcribed by the main researcher.

The analysis of the transcribed texts was carried out using a qualitative content analysis methodology [14]. Based on the bibliography studied in phase 1, four categories were defined: empowerment, perception of loneliness, psychological needs, variables influencing the process. Each interview was read by two members of the team and coded according to these categories. Since there were only four interviews, the process was carried out manually, discussing the results in periodic meetings. The text fragments presented in this paper were selected considering the above categories. Finally, we had the collaboration of an external judge and a social psychologist, who studied the consensus between the results and the categories proposed.

**Figure 1:**
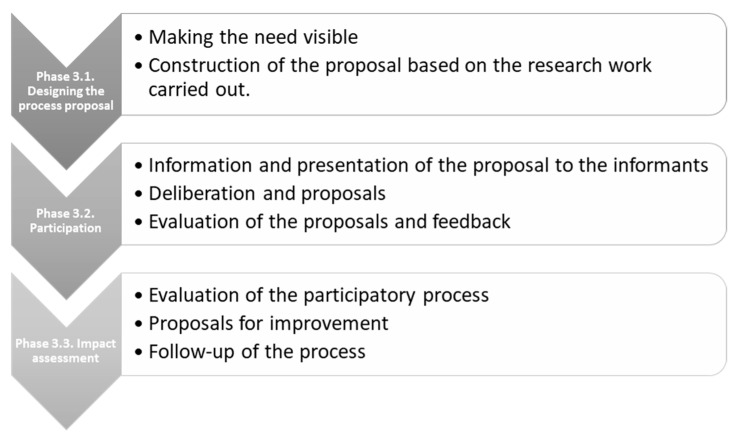
Phases of the participatory process. Own elaboration based on Government of Aragon [19].

According to specific objective 3, in the last phase, we proceeded to design the family intervention protocol, with a participatory methodology. For the design of this process, we took as a reference protocols and guides already existing in Aragon [15,16,17,18] and the methodology used for the design of its public policies by the Government of Aragon adapted as Figure 1 presents:

In view of the importance of the involvement of the people concerned in the research and the proposals that will emerge from it, the results of this process and previous interviews will be shown in the following paragraphs.

## 3. Childhood Cancer and Family: Current Situation

### 3.1. Contextualisation: Treatment and Sequelae

It should be clarified that childhood cancer is defined as cancer that appears before the age of 15 years [20]. Its incidence “varies between 50 and 200 per million in children and between 90 and 300 per million in adolescents” [21]. It represents “between 0.5% and 4.6% of the total number of cancer cases in the population” [21].

One of the differences to be highlighted between adult and childhood cancer is its etiology, being of multi-causal origin and practically unknown in the latter. Although in 5% of cases it is genetic, there are other environmental factors for which little information is available given the difficulty of knowing where they were found before they grew up [22]. For example, the oscillation of global incidence figures depends on the geopolitical and economic situation, being lower in Europe and North America than in Africa [23].

The survival rate (5 years after discharge) has increased over the last 50 years from less than 30% to more than 80%, being lower in countries with a lower human development index [20,21,22,23]. Despite this decline, the process is still generally long and hard, as informant 5 highlighted:

“This is a long treatment and it’s a hard treatment, from which you get out but you cannot run (...) Cancer is a word that yes, it gives horror, but cancer does not mean death, it means that you go through a lot of things but you can get out, you can come back.”

(Informant 5)

**Figure 2:**
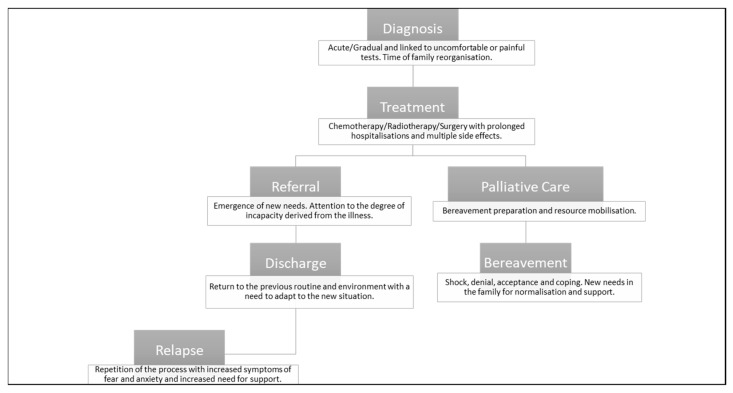
Disease phases. Own elaboration based on information from the Spanish Federation of Parents of Children with Cancer [13,24], ASPANOA, and Arenas [10].

Moreover, it is a process that goes through different phases (see Figure 2) with its own characteristics and needs, which vary according to different variables, which will be explained later on.

Each of these phases and its characteristics will vary the situation in which the family finds itself. Consequently, the professional relationship and communication with the patients and their families must be flexible at all times during the intervention [5,10]. Within these stages, the diagnosis is particularly complicated, because of the impact it has on the psychological level, the painful tests, social changes, and the hospitalization it entails. It is defined as a universal phase, and “one of the critical moments in the disease process” [10]. It is described by families as:

“The worst moment of my life was those days (...) I needed to catch my breath (...) I was totally gone and with the vision of my dead son”.

(Informant 4)

“I couldn’t believe it (...) you don’t know which way the air blows (...) I couldn’t find comfort anywhere.”

(Informant 5)

Childhood cancer diagnosis is made in a single hospital in Aragon, often kilometers away from the family home. The signs of suspicion of the disease can occur in three ways: in a routine check-up, due to discrete symptoms, or in emergency. These differences in symptomatology “influence the subsequent experience of the disease” (Informant 3) and “vary the waiting time for diagnosis, between 1 and 2 weeks in the first two cases and 1 to 2 days in the last” (Informant 2).

Once the diagnosis is confirmed, the doctor communicates it to the parents, who decide whether and how to communicate it to the child. Therefore, their role is fundamental, as it is positive that it is the family who communicates the diagnosis to the child, giving as much normality and optimism as possible, always adapting the information to patient’s demand and age (Informants 2, 3, 4 and 5). As Arenas [10] points out, it is advisable to have a systemic approach, considering the family environment. Considering that:

“All this leaves some very important after-effects and not only cancer, but all serious illnesses, leave family and personal after-effects which you have to be working all day long.”

(Informant 5)

### 3.2. Childhood Cancer Impact on the Family

Childhood cancer diagnosis changes the family dynamics in areas such as the emotional, socioeconomic and organizational. On an emotional level, as has already been mentioned, this disease has a greater impact than others. Emotional reactions go through different phases and are “very intense feelings of disbelief, anger, fear, guilt and grief, often accompanied by tendencies towards isolation and withdrawal of the family into itself” [25].

These feelings are psychologically healthy and adaptive in the first moments of a crisis situation such as the one we are dealing with. On the other hand, if they persist and affect the normal functioning of the family or any of its members, the psychosocial approach must be modified. Some of the emotional consequences of the diagnosis will not disappear during the disease course, nor in moments after recovery, such as fear of relapse [5,6,12,26]. These should be considered when intervening and communicating with families in order to avoid problems such as a hostile environment, tyranny on the part of the sick, transferring fears to children, lack of confidence or limiting activities [24].

Within coping, special attention should be paid to the relationship between stress and cancer, as the diagnosis brings uncertainty and harm to the child and thus a loss of control [27,28]. At the time of diagnosis:

“What worries you most is what is going to happen tomorrow, what am I going to do with my work, what am I going to do with my other children, how long is this going to last, and that is something you’ve to learn to focus on (...) They told me not to ask so many questions, because this may or may not happen, but I wanted to have everything under control, because when I have everything under control it seems like you know how to handle it, but when I do not, I have learned that you cannot control everything.”

(Informant 5)

In this sense, the way these situations are dealt with will influence the experience of the patient and their family, their understanding of the disease and, therefore, issues such as adherence to treatment or the return home after discharge from hospital [28]. It is suggested that adequate social support allows for greater stress management and improved quality of life for patients. Therefore, therapeutic intervention, the maintenance of a state of playfulness, physical and cognitive activities, as well as the creation of support groups are essential to prevent possible sequelae in mental health [3,5,10,12,24,29].

“I do not think he wasn’t aware, while he was in treatment he was not aware, of course he was 5 years old and wasn’t aware. He went to the hospital to play, in fact, he was more at ease than at home (...) Afterwards, yes, he himself says “how lucky I was to be as old as I was and not find out at the time” (...) I miss the availability I had when I was in hospital: I was always ready to play, I was never tired, afterwards I didn’t do it, not before either, I will value that year all my life.”

(Informant 4)

“Communication with families is important so that the child knows what he/she has. We have come across, for example, a case of a 17-year-old who thought that what he had was appendicitis, and no, it was cancer.”

(Informant 3)

The relationship of trust with the professional plays an important role, so that it not only favors communication but also helps in the understanding of the situation and the search for information that comes from the professional sphere and supports the needs of the child [5,6,12,24,28,30]. Families comment:

“They transmit confidence, the medical team, from nurses to everyone, they transmit confidence and it’s fundamental. (...) there are children who go to the hospital crying, too, but the fewest, and little by little you see a total change in them, of at first total rejection and then assimilating it and in the end, I go because they are not going to do anything to me that they don’t have to do to me. It is up to them to transmit that. (...) And parents, too, they tell us “whatever I have to tell you I will tell you whether it’s good or bad”, so you no longer go with fear.”

(Informant 4)

To this impact must be added the socio-economic difficulties inherent to hospitalization and treatment, such as: work absences, travels, family reorganization, reduced time for oneself, or the vulnerability of other relatives (children or dependent relatives, for example) [5,12]. Special consideration should be given to this last point, trying to minimize the impact that this new situation may have on siblings and preventing them from feeling displaced [2,5,6,24,28] (Informants 2 and 3). The work that can be carried out by the family environment is also important, especially in countries such as Spain:

“The grandparents are a fundamental pillar because if the mother or father has to be replaced because they have to work, there is the grandmother; if they have to cook, there is the grandmother; if the day you leave the hospital you don’t have time, there is the grandmother. Even if she is not here (in the hospital), because I had my mother here with me, but I had my mother-in-law with my little girl, if my mother-in-law had not been here I do not know what would have happened. I would have had to be with my mother and I would have had to be alone.”

(Informant 5)

Addressing these problems is directly related to the objective of oncological social work and, with the conception of the person as a biopsychosocial being, “so that they can cope in better conditions both with their current problems and with other conflictive situations that may arise in the future” [31]. Considering, furthermore, that the families with whom we are going to intervene “also bring with them their family history” [9], they must be attended to in a personalized and flexible way. To facilitate this, we identified some variables that affect the impact described on Table 1, which should be considered in the intervention:

Within these variables, the importance of the support network and its awareness of the disease stands out, as its reaction will influence the trust that the parents place in the child,

“You need someone to pick you up and take care of you and pamper you and you can talk so that you can recover and help your child and the rest of the family, which is important (...) Communicating it to the rest of the people around you, not the closest people, was complicated, they were not very close but they were not strangers either. They would ask you and, when I told them, I remember that the face of the person in front of me would change completely, that face was the one that killed me (...) There came a moment when I decided” “I am not going to tell, when I can, I will tell”.

(Informant 5)

For this, it is important to highlight the relevance of awareness-raising, information and social sensitization work in parallel to family intervention.

### 3.3. Family Intervention in the Childhood Cancer Diagnosis

The purpose of social work is for those affected to undertake a process of self-knowledge and growth that will help them to alleviate current and future stressful situations [5,31]. It is worth noting that, according to the SSWLHC [32] standards, there should be at least one social worker available 24 h a day, 7 days a week, in the hospital, with the possibility of telephone support.

Despite this, the reality in Aragón (Spain) is quite different. First of all, there is only one social worker from Monday to Friday, in the mornings, for all of Miguel Servet Maternal and Child Hospital. In addition, this hospital treats people from Aragon, La Rioja, and some cases from Soria, covering three different Spanish regions, attending in 2019 to 47,000 emergencies, 7050 admissions and 62,800 consultations. In this sense, the health care and social service systems allow for public–private collaboration that supports the work of their professionals, involving civil society, as is the case of ASPANOA and Miguel Servet Hospital. In this case, ASPANOA staff is present in the onco-pediatric unit all week, including a social worker and a psychologist.

The demand for support may come from the family itself or from the health staff. Therefore, the information given in the initial interview should clarify the professional role in the process. The study can be supported by the data provided by the clinical history, such as the place of residence or the existence or not of previous illnesses. In turn, as this is specialized care, there will be some basic needs related to the disease that can be addressed with greater foresight, such as school absences or the need for a caregiver in hospital [2,5,12].

Attending to the diagnosis, the present protocol is approached from the perspective of the crisis intervention model, given the rupture that this represents for the patients and their families. The moment of crisis is defined as that caused by an accidental event, in which what could be termed as difficulties that could be resolved without help and which affect individuals to varying degrees are exceeded. Based on the crisis phases, this specific case, and the principles of the Crisis Intervention Model [33,34], three fundamental tasks can be defined: “Realistic perception of the event and the feelings it arouses; Searching for adequate support in the environment; Putting capacities into action” [33].

**Figure 3:**
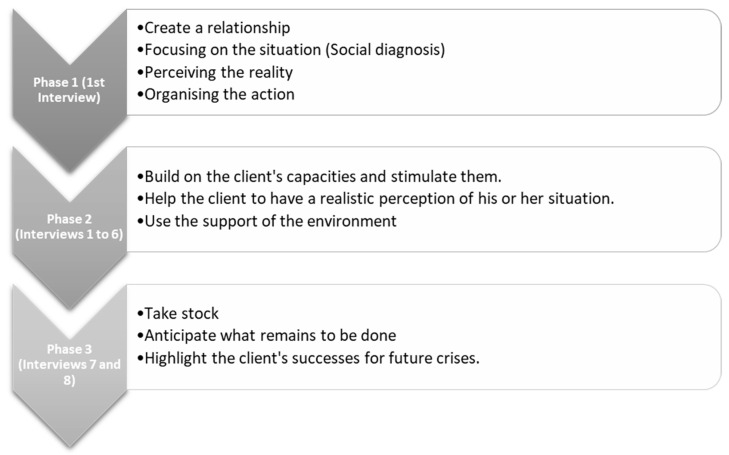
Intervention phases. Own elaboration, based on Du Ranquet [33].

These tasks will be broken down into three phases [34] (see Figure 3), which will be further broken down into different sub-phases of the intervention procedure:

According to this model, it should be noted that there are profiles for which this model will not be useful, and which refer to people who live in a chronic state of crisis, i.e., who are in a state of continuous exhaustion and who require long-term interventions, using other models, such as ecological or systemic models and their protocols, for example, people in a borderline state, whose option is flight; or people at risk of exclusion or who lacked adaptive resources before the crisis [33]. This will be another of the variables to be added to those presented above.

Social work intervention can facilitate adaptation, offering information to the family for decision-making, always respecting its dynamics and structure [5,6,12,28,35]. Bearing in mind the needs identified by the families as the main needs for coping with the illness—freedom, trust, support and conducting activities outside the hospital (Informants 4 and 5)—the relevance of the relationship of trust and communication should be highlighted.

As far as the relationship of trust is concerned, as studies indicate, “it also has to do with the level of general satisfaction perceived during their stay in hospital” [24]. In this sense, seven agents can be defined to be considered in the relationship of trust: (a) hospital center; (b) medical staff, their proximity and accessibility; (c) nursing staff, from whom a sense of control and security, respect for privacy and state of mind is demanded; (d) psychologists; (e) teachers; (f) volunteers; and (g) social workers [24].

An important issue in relation to these agents is the emotional exhaustion involved in care work, which is greater in onco-pediatric than in other specialties [6,36,37]. For this reason, it is essential to work on the professional care itself to avoid burnout, thus improving patient and family care. Thus, various publications have proposed the need for supervision and support, involving the people responsible for social and health care [4,5,6,32,36,38].

In terms of communication, there are demands for affection, empathy, trust, closeness and language adjustment (Informants 4 and 5). Consequently, we propose to use the Setting, Perception, Information, Knowledge, Empathy, and Summarize (SPIKES) protocol for the communication of bad news, which considers the situation and diagnosis repercussions in terms of stress and attempts to reaffirm an empathetic relationship, also improving trust [39].

In onco-pediatric cases, it has been shown that diagnosis communication to these patients obtains a better response if it is given in a realistic and age-appropriate way [5,8,30]. In the same study, the news communication to both patients and relatives was even considered under the following argument: “Doing this shows respect for the child as an individual and as a patient and, as one seven-year-old girl said, made sure that “everyone knows” (...) Receiving bad news together also provided an opportunity to (...) support each other” [30].

Despite this argument, there are some dilemmas: it is important that it is delivered in a way that is adjusted to reality, maintaining hope and counting on the family, although it is understood that the family may affect the patient if it is not channeled. In this sense, the professionals propose that:

“Always tell the truth and let the parents break the news to the child, allowing them to ask. The process would be more or less: the doctors tell the parents, who try to cross-check the information, and then they should tell the children.”

(Informant 3)

To this end, the professionals will give keys to the family to enable them to communicate effectively, knowing that they will have to consider both their emotional situation, which will allow them to communicate in the optimistic way demanded by patients, as well as issues related to beliefs, concerns, expectations and non-denial of their feelings [24]. In this sense, families highlight the importance of seeking support from professionals to clarify the doubts of children and provide keys to communication (Informants 4 and 5).

In terms of the resources available to families, there are many and varied resources, highlighting that there are differences in those benefits not recognized by state-level laws and which generate inequalities, given the Spanish autonomous communities structure. For this reason, the patient origin or the existence or not of associations that provide some service where the hospitalization takes place must be considered.

## 4. Proposal for a Family Intervention Protocol for Comprehensive Care at the Time of Childhood Cancer Diagnosis

This intervention will be based on the following basic principles [2,4,5,17], which will be added to those of the Crisis Intervention Model:

1. Adapt to the patient reality, with the best interests of the child prevailing;
2. Respect the individuality of the relatives;
3. Enhance the capacities of parents, guardians or custodians;
4. To enhance the child self-protection and resilience factors;
5. Networking.

In accordance with the model chosen, this protocol has been designed to last 8 weeks, and can be extended between 1 and 3 weeks depending on the time it takes to find the appropriate diagnosis and treatment, as well as the psychosocial family situation. For this, coordination between professionals from the Miguel Servet Maternal and Child Hospital onco-pediatric unit [40], ASPANOA, and volunteers, especially “veteran families” who come to support the first impact, will be essential.

With regard to the professional competencies that should be strengthened for the application of this protocol, those determined by ASPOW [4,5,6] and related to social skills: silence, active listening, empathy and assertiveness [41]; and communication of bad news and bereavement support stand out: clinical, educational, legal, research, ethical and professional, and listening and understanding, using the SPIKES protocol [2,4,5,32,39].

**Figure 4:**
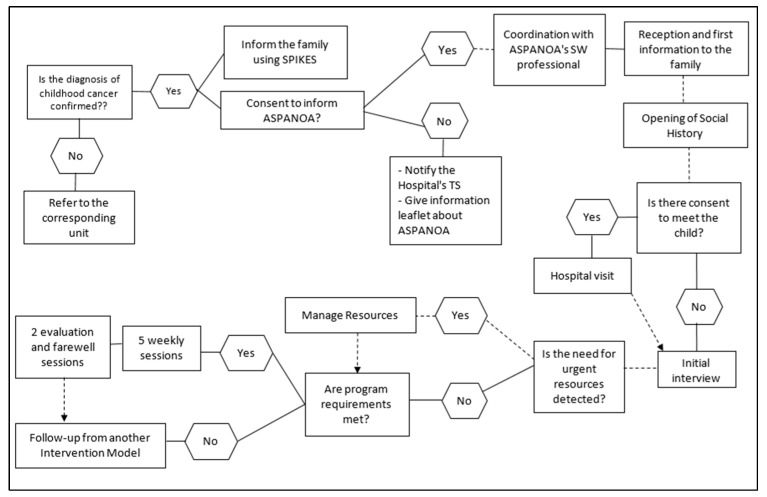
Algorithm for action.

The development of the social worker’s functions will be conducted according to the following scheme, being flexible and appropriate to the moment in which family and illness are variable over time [2,4,5,10,28] (Informants 2 and 3) (See Figure 4):

1. Coordination. The referring doctor will coordinate with ASPANOA through the Hospital social worker, who will request ASPANOA’s collaboration for support after the diagnosis.

This will provide details of the medical diagnosis, treatment plan and prognosis (if any) and basic information about the child and his or her family. In turn, the social worker will provide information on possible social risks and preventive actions to be conducted jointly, always with the prior family authorization.

2. Reception and initial information for the family. The doctor introduces the social worker to family. Through the presentation of the entity as a resource and the delivery of informative material, first contact will be made with the family that will allow a relationship of trust to be established with them. The social history will be opened.
3. Hospital visit. Presentation with the child, if the family gives their consent.
4. Initial interview, which will be carried out as soon as the parents allow it, trying to make them feel welcome but not overwhelmed by the information. The objectives will be:
  a. Deepening the relationship of trust, emotional support and accompaniment.
  b. Establishing the social, work, economic, school and family situation.
  c. Identifying the main risk indicators [2,5,12,28,29] (Informants 2 and 3):
    - Lack of resources (personal skills, language problems, economic deprivation, lack of support, etc.);
    - Need for accommodation;
    - Altered roles and relationships in the family environment;
    - Need for diet for caregivers;
    - Employment parents’ difficulties (exploring moonlighting or no possibility of taking leave to cover care needs);
    - Child school situation (exploring possible conflicts);
    - Existence of relatives with an illness/disability;
    - Existence of other children at home;
    - Overburdening of caregivers;
    - Family experiences of traumatic situations;
    - Experiences of health neglect;
  d. Identify family and child protection factors.

After this, the data will be analyzed and interpreted in order to draw up a social diagnosis.

5. Association resource management and coordination with the professionals involved, in order to pool the factors that need to be taken into consideration when dealing with the disease. Coordination with ASPANOA volunteers so that they can support the family.
6. Five weekly family follow-up sessions, which will be distributed according to the family situation and the illness, and some of the sessions may be devoted solely to emotional support and containment. These sessions will address the following issues:
  e. Fostering a relationship of trust and clarification of doubts.
  f. Attention to latent needs that have not previously arisen. Follow-up of the resources provided and evaluation of their usefulness.
  g. Addressing diagnosis communication and support to the extended family, the child and siblings (if any), and the support network, such as grandparents. Tools and strategies will be offered to facilitate this communication, strengths will be promoted and support will be given in answering possible questions. The resolution of doubts through the healthcare team will be recommended.
  h. Support in family reorganization and decision-making based on co-responsible participation, with the child and their siblings, if any.
  i. Family intervention with a view to adapting to the new reality, maintaining behavioral guidelines and adapting rules. Special attention should be paid in the event that there are more children in order to avoid possible conflicts or harm to them.
  j. Sustenance for relations with the support network and maintenance of leisure and recreational spaces for the parents, favoring spaces for self-care and distraction, with the possibility of using voluntary resources.
7. Conducting two weekly interviews aimed at closing the intervention, evaluation and preparation for a follow-up during the disease process, bearing in mind that the work will continue using other intervention models. It should be made clear that an ASPANOA social worker and psychologist will be at the hospital and at the association’s headquarters at the family’s disposal, monitoring and accompanying them throughout the illness, helping the hospital social worker. Their main objectives will be:
  k. Take stock of the reaction and changes experienced.
  l. Highlight the positive and reinforcing elements detected.
  m. Prepare for future changes or crises throughout the illness and its aftermath.

Bearing in mind the interdisciplinary nature of care, networking must be maintained throughout the illness course, with the ward team, the hospital teacher, the school where the child was attending, and any other resource with which the child has previously had contact. In this respect, a monthly coordination meeting is held between the Hospital and ASPANOA professionals. Educational matters are dealt with by the school and the hospital or home care teacher, while those related to other social resources are dealt with by the social worker.

This coordination and networking will also be used for supervision and mutual support between professionals, both educational and emotional [5,6]. These sessions are flexible and designed with the professionals, with the main objectives of avoiding burnout and improving the intervention. If deemed necessary, they may be led by external professionals addressing specific topics [32,36,37,38].

Finally, we propose three evaluations through systematically collected data:

1. Coverage assessment: how many families have been served and what are their characteristics.
2. Evaluation of process: intervention carried out and resources used.
3. Evaluation of results such as: degree of coverage, problem solving, resources used, family evolution or reason for termination of the intervention.

## 5. Conclusions

### 5.1. Situation Diagnosis

Firstly, the lack of Spanish research in the field of onco-pediatric social work should be highlighted, in spite of the amount of scientific literature about it in health sciences. There is a lack of focus on the need to generate protocols of action adaptable to each individual in the field of social work in comparison with other professions and health contexts, such as the USA [4,5,6]. Because of that, we agree with Wiener [6] on the need to define social work as an essential and health profession, which is not yet a reality in Spain.

Secondly, thanks to the search for and comparison of existing resources, inequalities have been detected in access to them, depending on the Autonomous Community of children’s residence or treatment [13]. According to various authors [4,5,6,9,10,11,12,21,23,35], this gap should be reduced by universalising and standardising access throughout the country. Based on the interviews carried out, the automatic granting of at least 33% disability at the time of diagnosis for children affected by serious illnesses, such as childhood cancer, revisable 5 years after discharge, is proposed as a measure. This would allow access to state and/or regional benefits that would provide the necessary economic and social support for families.

This measure would support the deficits detected in the interviews in the categories of coverage of psychological needs [5,7], reduce some of the variables detected and improve the perception of institutional support [7,8].

Finally, despite the fact that the survival rate is increasing, society maintains a perception that relates cancer to death, generating a feeling of pity. For this reason, awareness-raising actions are needed that favor the de-dramatization of cancer, giving it the importance it has but allowing those affected to go on with their lives as normally and with as much support as possible. This supports the categories of empowerment [7,8], psychological needs [5,7] and perceptions of loneliness, increasing community support [7,8].

### 5.2. Coping with Childhood Cancer by Those Affected, Their Family and Close Environment

Firstly, as several authors have commented [5,12,24,26,29,30], there is a relationship between the family’s coping with childhood cancer, communication with the child and with the health and social care team. These factors influence disease-related issues such as adherence to treatment [1,5]. Therefore, it is essential to establish a protocol for the communication of bad news and for the reception of families by professionals to prevent possible sequelae in the mental health of the patient and family, such as the one proposed by Kaplan [39].

According to the interviews carried out, the role played by the family’s agents and support network, such as grandparents, who, in addition, due to their age, tend to be more vulnerable, should be borne in mind when carrying out a good reception and coping process. In this sense, it is necessary to intervene with them through social work to address their demands and their role in the new family dynamics, as some authors have argued before [7,8,25].

Thirdly, in accordance with the variables studied and the changes in family models in recent years, the incorporation of a mediation service in the health system is recommended. This could orient towards the distribution of roles and new tasks in the new situation, and attention to possible conflicts derived from separations, which could interfere in the coping with the illness, affecting the child. It could be extended to realities such as people in a situation of supervening dependency [5,7,9,10].

### 5.3. Proposals to Develop

Firstly, according to the literature studied [4,5,6,23,31,32,35], the creation of protocols with the actions to be carried out during the illness reaffirms the quality standards in the intervention. In this sense, the participation of the agents involved and affected by the disease (social and health care team, families, patients, organizations, volunteers, etc.) is necessary in the preparation of this type of document, thus favoring a greater adjustment to their reality. We stress the need for flexibility when developing this protocol, considering the situation at all times and leaving space for reflection and assimilation [6].

In turn, our results show that transversality and interdisciplinarity are very present throughout the intervention, highlighting the importance of coordination. In addition, several authors have detected a high risk of burnout in onco-paediatric teams [4,6,32,36,38]. Hence, it is necessary to create a specific supervision program, such as that of Puig Cruells [38], to promote awareness, the sharing of cases, prevention, and support and training of the care team, as well as generating support material in the form of guides.

Finally, we are aware of the limitations of the sample analysis, as this is a pilot study focused on one autonomous community. However, our aim is to give voice to the needs of this group and the relevance of social work in alleviating the effects of the disease on their quality of life. Despite these limitations, we believe that our pilot protocol could be further applied in research of larger territories or samples, other serious illnesses such as eating disorders or premature babies, or even the study of the long-term effects of its application. It could also be adapted to other moments of the disease, considering its special casuistry, such as palliative care.

## Figures and Tables

**Table 1:** Variables to consider in family intervention.

In the Patient	In the Family	Common to Both
Patient’s age and development	Family situation (Description of the family situation, typology, existence of pregnancies, special attention to single parents)	Culture and language, especially for immigrant families
Educational status	Siblings: Existence or not and age	Previous experiences and emotions/grief situations
Existence of previous disorders or health problems aggravating the disease	Rural environment and/or necessary displacements	Previous experiences of traumatic situations, especially health or lack of resources
	Intra-family conflicts	Length of hospital stays and prognosis
	Socioeconomic/employment/health situation of the family	Personal resources
	Extended family: Existence or not and relationship to extended family	Support networks: resources and help from the environment
	Caregiver overload and change of roles	Culture and language, especially for immigrant families
	Health of family members	
	Family situation (Description of the family situation, typology, existence of pregnancies, special attention to single parents)	
	Siblings: Existence or not and age	
	Intra-family conflicts	

Own elaboration based on Informants 2 and 3 [5,6,9,10,12,28,32].

## Data Availability

Not applicable.
